# On-Line Estimation of Laser-Drilled Hole Depth Using a Machine Vision Method

**DOI:** 10.3390/s120810148

**Published:** 2012-07-27

**Authors:** Chao-Ching Ho, Jun-Jia He, Te-Ying Liao

**Affiliations:** Department of Mechanical Engineering, National Yunlin University of Science and Technology, Douliou, Yunlin 64002, Taiwan; E-Mails: g9911733@yuntech.edu.tw (J.-J.H.); D10011002@yuntech.edu.tw (T.-Y.L.)

**Keywords:** machine vision, on-line estimation, laser-drilled hole depth, laser drilling, laser machining

## Abstract

The paper presents a novel method for monitoring and estimating the depth of a laser-drilled hole using machine vision. Through on-line image acquisition and analysis in laser machining processes, we could simultaneously obtain correlations between the machining processes and analyzed images. Based on the machine vision method, the depths of laser-machined holes could be estimated in real time. Therefore, a low cost on-line inspection system is developed to increase productivity. All of the processing work was performed in air under standard atmospheric conditions and gas assist was used. A correlation between the cumulative size of the laser-induced plasma region and the depth of the hole is presented. The result indicates that the estimated depths of the laser-drilled holes were a linear function of the cumulative plasma size, with a high degree of confidence. This research provides a novel machine vision-based method for estimating the depths of laser-drilled holes in real time.

## 1. Introduction

The development of laser-based machining has been ongoing for a long time and is applied to all aspects of conventional industry, semiconductor manufacture, the biochemical and biomedical sectors, *etc.* Because lasers use very high energy, they have always previously been applied in conventional cutting and ablation processes. More recently, lasers have gradually also been applied to precision micromachining processes. The high energy produced by a laser makes precision control and monitoring difficult in micromachining. Therefore, a laser machining system that utilizes pulse laser machining rather than continuous laser machining has been developed. A pulse laser can be controlled more accurately than a conventional continuous laser system. However, the control is still not sufficient to apply to micromachining. In recent years, various measuring methods and technologies have been researched for laser machining monitoring and control systems, including electromagnetic fields, electric fields, acoustics, optodynamics, and optics.

### 1.1. Patent Research

In 1991, Koegl *et al.* disclosed a patent for a breakthrough detector that used magnetic field pickup coils placed upon the surface of a workpiece for the real-time detection of the magnetic field signal of laser-induced plasma during laser drilling. This method could detect whether the laser-drilled hole perforated the workpiece. The method claim to detect the depth of a laser-drilled hole in real time [1,2].

### 1.2. Acoustic Research

In 1996, Stauter *et al.* found that a short pulse laser generated shock waves during laser drilling. The shock wave energy reflected from the laser-machined region was detected using the laser beam deflection and a microphone. They demonstrated a system for the real-time control and measurement of the laser machining process [3]. In 2003, Palanco *et al.* presented a similar method of using a microphone to acquire the shock wave from the laser machining process and analyzed the spectra of the laser-machined shock waves of various metal materials in the time and frequency domains. Their experimental results showed a correlation between the spectra of the shock wave and the laser-induced plasma, but an accurate correlation was limited by the measurement configuration and equipment [4]. In 2010, Stournaras *et al.* established an experimental setup and performed a theoretical analysis to investigate the acoustic signal from laser drilling. The experiment results showed a good correlation between the signal's amplitude and the hole's depth based on the flow of gas-assisted machining, but it could not precisely estimate the depth of a laser-drilled hole [5].

### 1.3. Optodynamic Research

In 2002, Strgar *et al.* presented a real-time method for determining the depth of laser-drilled holes during laser drilling. The developed setup could detect the laser-induced ultrasonic waves in the workpiece and in the surrounding air. Because the depth of a hole increases with the machine time, the propagation times of the laser-induced ultrasonic wave traveling from the bottom of the hole to the bottom of the workpiece could be measured by a piezoelectric transducer at the bottom of the workpiece. Then, based on the propagation speed of an ultrasonic wave in various materials, the depth of the laser-drilled hole could be detected in real time [6,7]. In 2005, Stafe *et al.* used the same experimental method to research the depths of laser-drilled holes in various materials [8].

### 1.4. Electric Field Research

In 2002, Bredice *et al.* found that a pulse laser generated a discharge phenomenon in an electric field during laser machining. They placed the workpiece in the middle of two plates connected to an RC circuit, and the laser-induced plasma induced the discharge phenomenon. The quantum of the discharge signal of the RC circuit was then used to obtain a signal related to the plasma energy. This experiment measured the plasma energy successfully [9]. In 2007, Bredice developed a real-time measurement system that resembled the above measuring setup. By calibrating the curve of the experimental results, the system could predict the temporal evolution of the electron density of the generated plasma in real time [10]. In 2004 and 2008, Madjid *et al.* established another measurement mechanism which had two flat electrodes arranged in parallel, with a gap that allowed the plasma to be sandwiched between them. The machining region included the workpiece and flat electrodes placed in an acrylic chamber filled with air and He gas. By supplying a DC voltage to the flat electrodes, the induced current of the laser-processed plasma was detected using an oscilloscope. In addition to measuring the current of the laser-induced plasma, it also could detect whether the laser penetrated the target [11,12].

### 1.5. Optical Signal Research

In 2007, a method utilizing the detection of shock waves generated during the interaction between laser beam and irradiated material was presented by Walter *et al.* [13]. However, this method is not suited for detection of breakthrough unless the rear side of the workpiece is also monitored. It is not easy to deploy sensors for such a rear side configuration in many applications. Also, schlieren photography was employed to visualize the air flow, and resulted in the machining process was not assisted by an air jet to avoid image acquisition problems. The air jet function can aid in a cleaner-looking cut and enhance the material removal rates.

In 2010, a real-time measurement system based on the correlation between the optical signals of the laser-induced plasma from the processing region acquired by photodiodes and the geometrical characteristics of the laser-drilled hole was presented by Stournaras *et al.* [14]. In 2010, Lin *et al.* developed an optical measurement system based on the confocal principle. This system could precisely measure the depths of microholes during laser drilling in real time; the system achieved a sensitivity of 0.5 μm [15].

**Figure 1.**
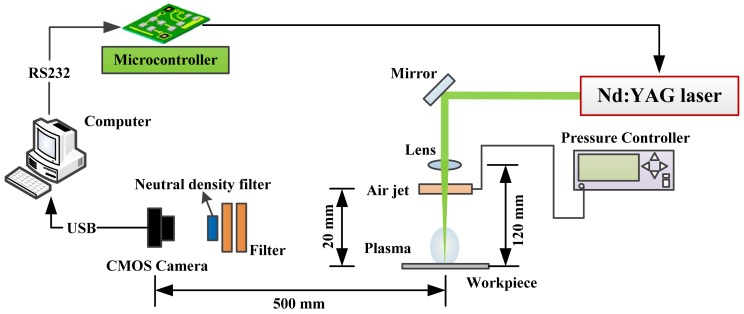
Experimental setup.

In this paper, we present a novel measuring method for the on-line monitoring of laser-drilled holes based on machine vision analysis. Therefore, on-line optical inspection system with low cost is developed to increase productivity. The monitoring of pulsed laser-induced plasma is used as the basis for on-line inspection control system for the hole drilling. Our measuring setup includes image acquisition units, laser machining equipment, and a computer analysis unit, as shown in Figure 1. First, we use the CMOS camera of the image acquisition unit for the on-line acquisition of an image of the laser machining zone of the workpiece's surface. The high laser energy will cause melting and vaporization of the metal material during laser machining, and cause the air and metallic vapor to ionize simultaneously. This ionization phenomenon will involve plasma and light, and it is captured by the CMOS camera. The images consist of both plasma and light information. Finally, the image data are sent to the computer analysis unit for an on-line analysis of the correlation between the geometrical forms of the machining zone, including the depth of a blind hole, and the image information. This experimental setup achieves the advantages of a lower cost, on-line monitoring, and good performance.

## 2. Experimental Setup and Method

### 2.1. Setup

The experiment setup, including the laser machining system, image acquisition unit, and computer control and analysis system, is shown in Figure 1. The computer is connected to a microcontroller, which controls the laser machining parameters, including the machining frequency and time, to trigger and control the laser machining system (LOTIS, LS-2134UTF, Nd:YAG laser system). The laser beam strikes the two kind of workpiece (aluminum, 5052, 0.6-mm thick, and stainless steel, 420, 0.8-mm thick) surface and induces the ionization phenomenon caused by plasma light. The plasma image of the ionization phenomenon passes through the wavelength filter (190–534 nm OD: 6.5, 850–1,070 nm OD: 5, 910–1,070 nm OD: 6) and neutral density filter (transmittance 13%) and is acquired by the CMOS camera mounted radially 500 mm from the work site. The microcontroller unit triggers the laser system to generate a single laser pulse and simultaneously delays a short time to control the image acquisition unit, which captures a single plasma image of the laser pulse. The plasma light is too bright for image analysis. To distinguish between the ionization phenomena for the air and material caused by the laser-induced plasma and to reduce the maximum brightness of the image acquired by the CMOS camera, a wavelength filter and neutral density filter are placed in front of the camera. The light from the laser-induced plasma that passes through the neutral density filter will remain at the wavelength of the ionizing material. The image analysis in this study focused on the ionization of the material from the laser-induced plasma. The ambient conditions and machining parameters of the experiment are listed in Table 1, where the air jet pressure is 300 Kpa.

### 2.2. Estimation Method

**Figure 2.**
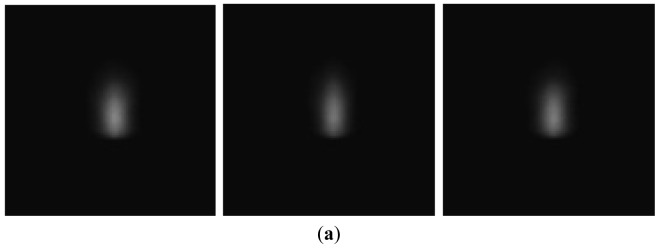

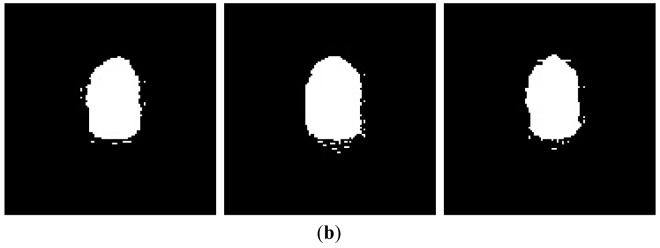
Plasma region of image of Al 5052 (**a**) acquired by CMOS camera and (**b**) after binarization.

**Figure 3.**
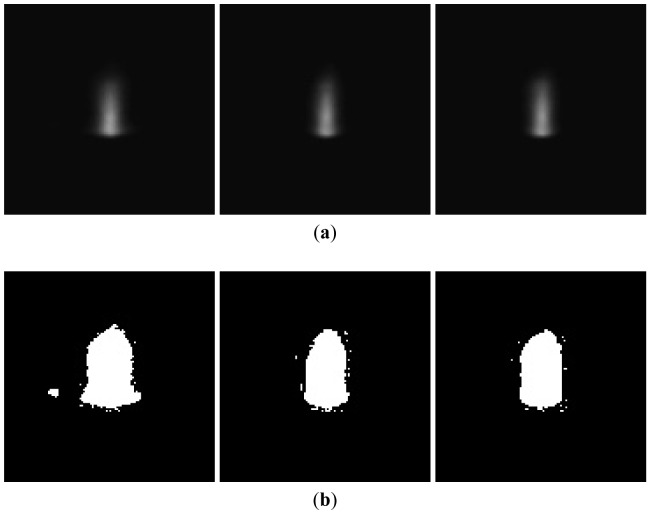
Plasma region of image of SS 420 (**a**) acquired by CMOS camera and (**b**) after binarization.

**Figure 4.**
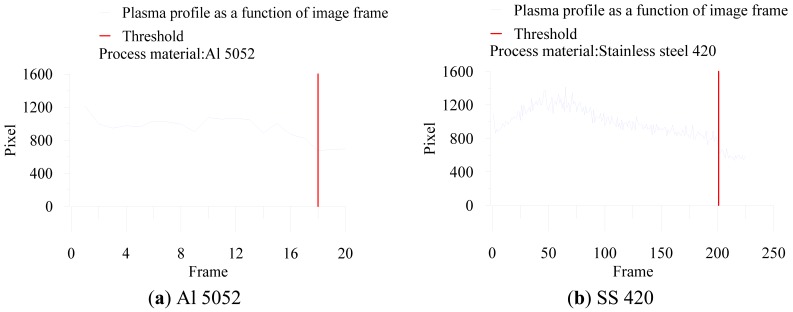
Breakthrough result based on abrupt decline in pixels of plasma region.

The laser-drilled machining, including breakthrough detection and blind hole depth analysis, were recorded and analyzed using 8-bit encoding and 120 × 120 pixel images. Through binarization during the image processing, we could determine the plasma emission region and calculate the total pixels of the plasma region. The images of aluminum and stainless steel after binarization are shown in Figure 2 and Figure 3, respectively. In breakthrough detection, the total pixels in the plasma region of a single image frame were recorded and analyzed. Breakthrough detection for laser drilling was successfully developed in a previous study [16]. Breakthrough detection can be briefly explained as follows. When a breakthrough occurs, the amount of plasma declines abruptly. The pixels in the plasma region of a single image frame would also decline abruptly and simultaneously at this time, as shown in Figure 4. Depth control and analysis for laser-drilled machining, the number of laser pulses is used to control the depth of a laser-drilled hole in the conventional control method. As the number of laser pulses increases, the depth of the hole would also increase. The conventional control method uses experience-based control rules to detect the completion of the ablation process off-line.

This machine vision method of finding the relation between the laser-induced plasma region and laser-drilled hole depth is a novel estimation mechanism for determining the depth of a laser-drilled hole for laser machining. During laser-drilled machining, a plume is usually produced as the result of vaporization of material from the laser heated area. The evaporated material is ejected and collides with energetic electrons and result in a rapid increase in the level of ionization within the plume with the formation of the plasma. The force induced by evaporated material, together with assist air jet, expelled molten material at the cavity to its outer rim [17].

The vaporization of all material removed is observed by the camera setup radially. Because it is difficult to find the relation between the laser-induced plasma region size and hole depth, the laser-drilled hole depth detection adopts a pixel accumulation method to estimate the hole depth, as distinguished from the breakthrough detection, which uses a single image frame analysis. The pixels of the laser-induced plasma region for each single image frame from the beginning to end of the machining are determined by using image processing. We then accumulate the total pixels for the single image frames of the plasma region during laser machining and obtain the total pixels of the plasma region from beginning to end.

## 3. Results and Discussion

In this study, an aluminum workpiece (aluminum, 5052, 0.6-mm thick) and a steel workpiece (stainless steel, 420, 0.8-mm thick) were used for the laser-drilled machining experiment, and the pulse laser machining system adopted a Nd:YAG laser (LOTIS, LS-2134UTF). The laser machining parameters are listed in Table 1. In these experiments, the drilling depth is determined after a couple of hours of careful grinding along vertical planes of the workpiece until the cavity is made visible. The depth and the diameter of holes and the thickness of recast are investigated by optical microscopy (OM).

**Figure 5.**
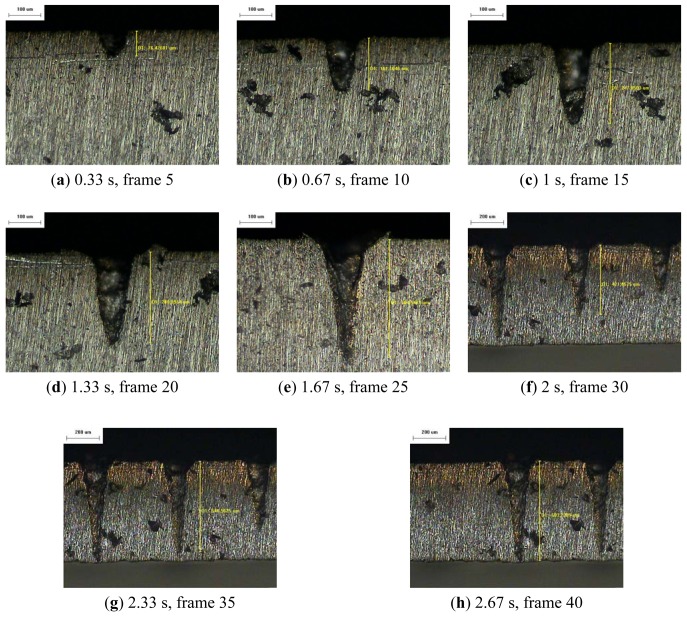
Workpiece (Al 5052) profile images at different process times (radiation energy is 145 mJ): (**a–e**) 100×, (**f–h**) 50×.

**Figure 6.**
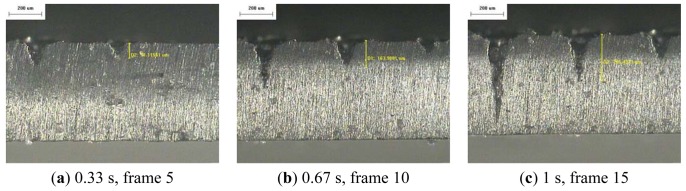

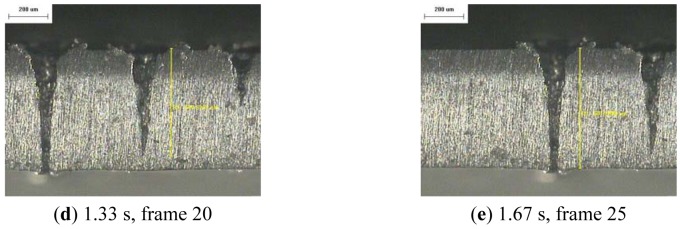
Workpiece (Al 5052) profile images at different process times (radiation energy is 175 mJ): (**a–e**) 50×.

**Figure 7.**
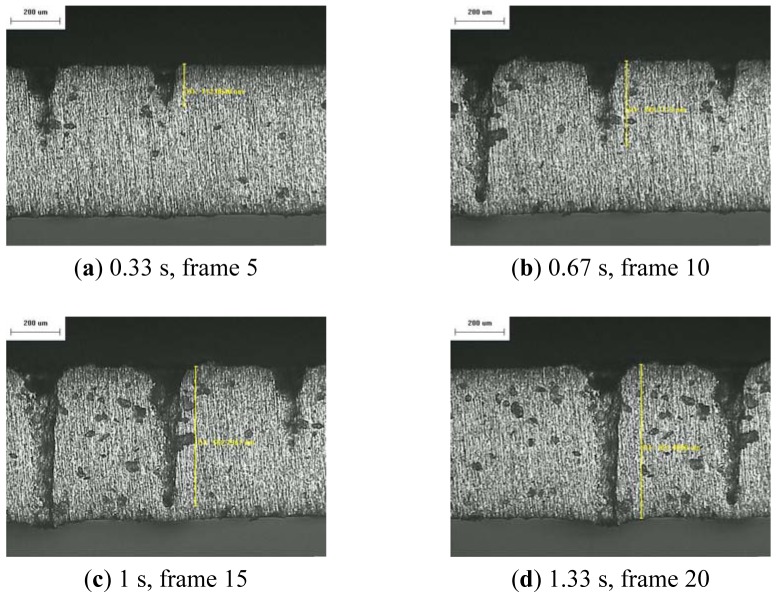
Workpiece (Al 5052) profile images at different process times (radiation energy is 200 mJ): (**a–d**) 50×.

**Figure 8.**
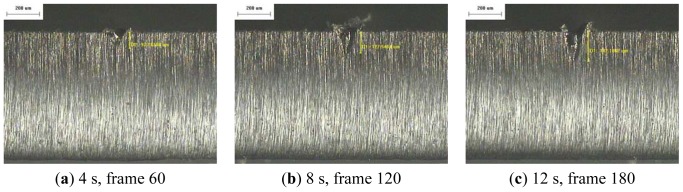

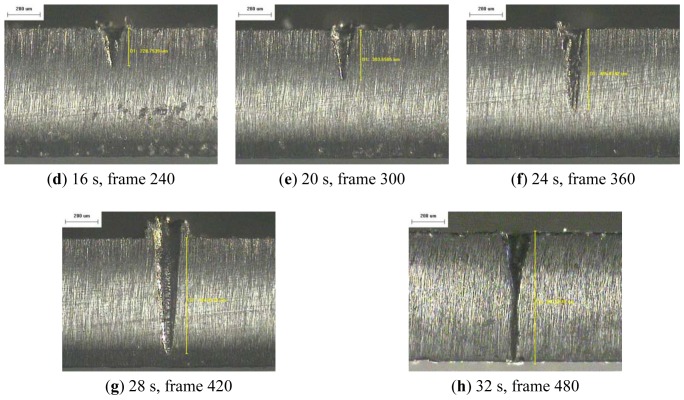
Workpiece (SS 420) profile images at different process times (radiation energy is 145 mJ): (**a–h**) 50×.

**Figure 9.**
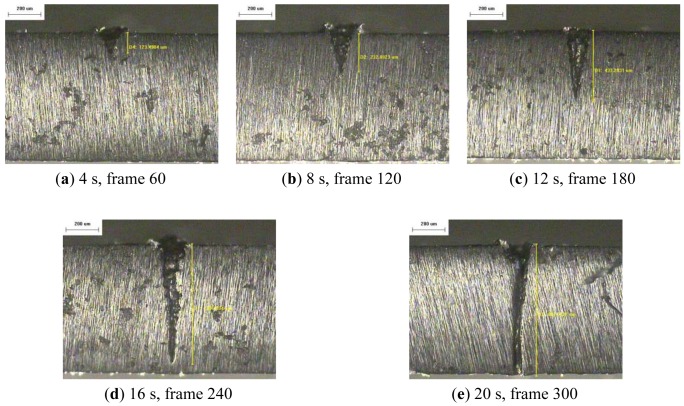
Workpiece (SS 420) profile images at different process times (radiation energy is 175 mJ): (**a–e**) 50×.

**Figure 10.**
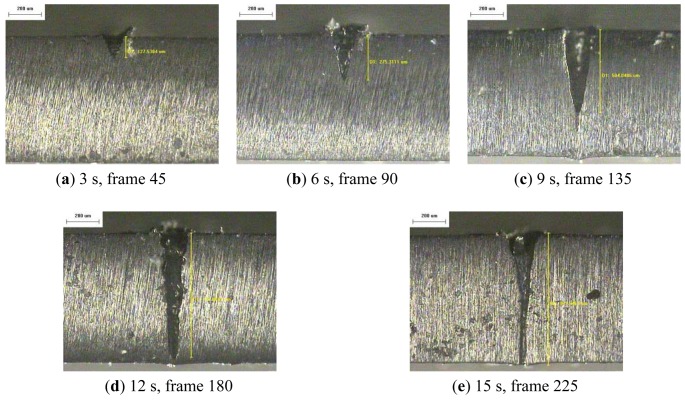
Workpiece (SS 420) profile images at different process times (radiation energy is 200 mJ): (**a–e**) 50×.

**Figure 11.**
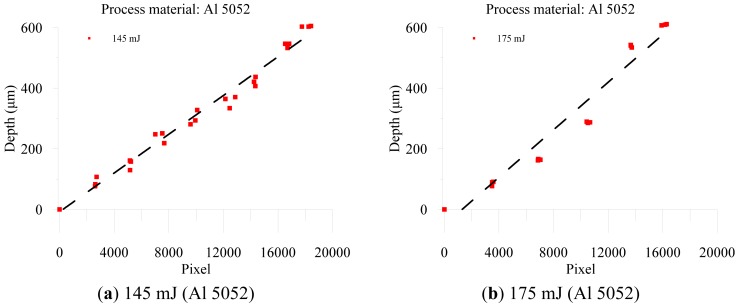

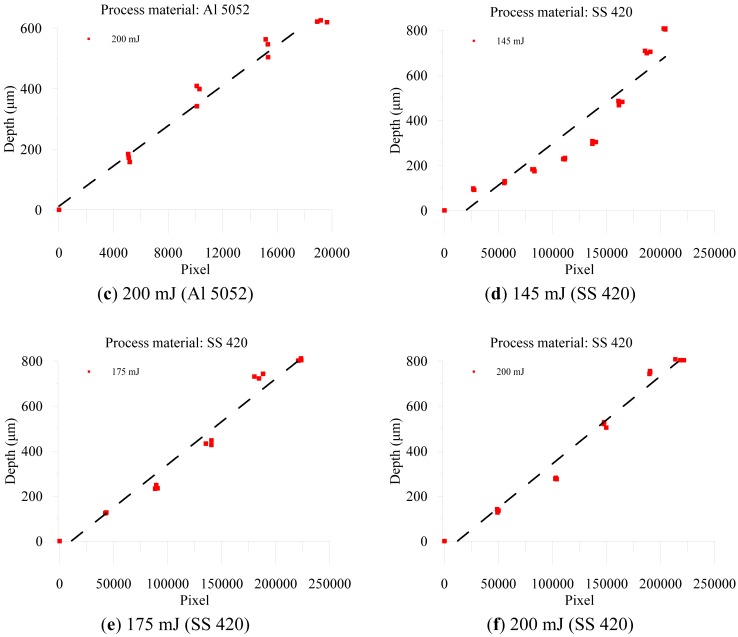
Relation between depth of laser-drilled hole and pixels of plasma region, at radiation energy of (**a**) 145 mJ (Al 5052); (**b**) 175 mJ (Al 5052); (**c**) 200 mJ (Al 5052); (**d**) 145 mJ (SS 420); (**e**) 175 mJ (SS 420); and (**d**) 200 mJ (SS 420).

The resulting profile images for laser-drilled machining using different process times, where the radiation energy of the pulse laser was 145 mJ, 175 mJ, and 200 mJ for aluminum and stainless steel are shown in Figures 5, 6, 7, 8, 9 and 10, respectively. It is obvious that the depth of the laser-drilled holes increases with the process time. The relation between the machining hole depth and pixel value of the laser-induced plasma region is shown in Figure 11. In all cases, the depth of the laser-drilled holes could be described by a monotonically increasing function, and for both materials the ablation depth rate increases with large laser energy. Since the heat transfer mechanism near the ablation zone was heavily affected by the thermal response of the materials to the incident laser pulses. The vaporization thermal properties is known to be affected by the material boiling temperature, which is lower in aluminum (T_vap_ = 2,740 K) than stainless steel (T_vap_ = 3,134 K), thus leading to an earlier ablation under the same incident laser pulses. There, a faster depth removal rate on the aluminum material than in steel material was observed at the ablation process as expected.

By investigating the depth of the laser-drilled hole as a function of the cumulative plasma size, we pave the way to fully control the process parameters. The depth estimated function *D* of the cumulative pixels of the total frame plasma region at the acquired ablation image frame *n* during laser machining is shown in Equation (1):

(1) $$D[n]=\alpha \sum_{i=1}^{n}A_{i}+\beta$$

where *A*_i_ represents the pixels of the plasma region of a single image frame, *α* depends on the thermal physical properties of the material (density, emissivity, thermal conductivity, specific heat, thermal diffusivity), and *β* depends on the laser energy. The higher pulse energies lead to the more plasma region *A*_i_, and hence a higher ablation rate can be expected. Same material under laser machined process reveals the same slope *α*. The higher laser pulse energy could ignite much more particle atmospheric plasma, and hence the parameter *β* depends on the radiation energy.

**Figure 12.**
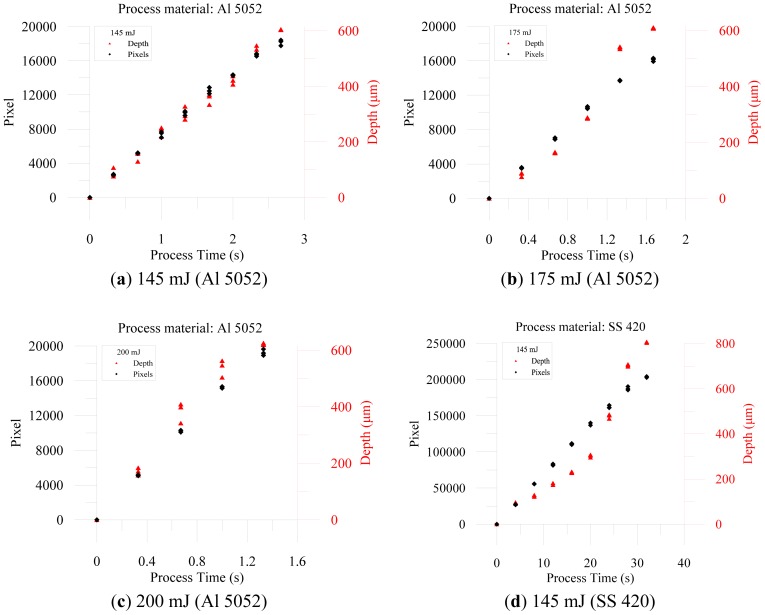

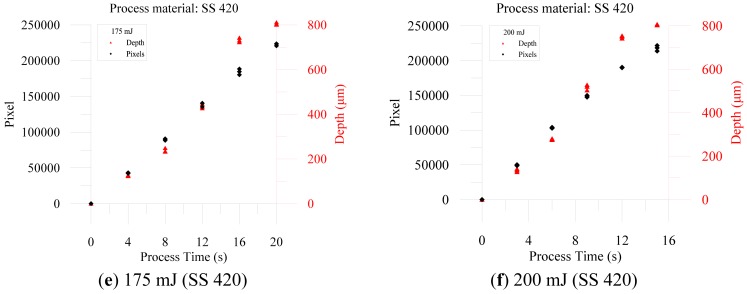
Relationship between pixels and depth, along with process time.

The results reveal that the cumulative plasma size has high coefficient of determination (*R*^2^) of 0.984 (aluminum, 145 mJ), 0.95 (aluminum, 175 mJ), 0.983 (aluminum, 200 mJ), 0.892 (stainless steel, 145 mJ), 0.975 (stainless steel, 175 mJ), and 0.978 (stainless steel, 200 mJ) with the machining depth, respectively. The hole depth is directly proportional to the machining pulses and the sum of the pixels of all the frames of the plasma region. Based on the proportional relation, we could use the total cumulative pixel values to estimate and detect the laser-drilled hole depth. Also, higher laser energy could induce both a larger region and additional pixels of plasma. Considering the fact that the radiation energy of the laser machining also affects the machining depth, a higher energy could generate deeper holes for the same process time, as shown in Figure 12. We also found that when the laser beam struck the material surface, the higher energy induced a larger plasma region. In this study of image processing based on machine vision, we determined that the size of the laser-induced plasma region at the surface of the workpiece could be transformed into the value of the pixels. The laser energy conditions determined both the laser-drilled hole depth and size of the plasma region, along with the process time. Thus, we could analyze and obtain the total pixels of the plasma region in on-line during processing, and we achieved a on-line detection and estimation of the depth of a laser-drilled hole. However, as shown in the Figure 12, the variations in the cumulative plasma size and the drilling depth with the time were observed.

These variations include the thermal properties of materials with temperature during laser processing, unexpected variations of about 2% in the laser output power, the ablation process also sensitive to surface finishing conditions, a confined plasma within deeper depths leading to a more thermal mechanism of ablation and finally, a reduction in laser irradiance due to changes in the effective area exposed to the laser beam. Also, for holes with high aspect ratios, the multiple beam reflections along the wall of cavity will affect the amount of absorbed energy and the increase the variations on accuracy of the depth estimation. Comparisons between different measurements show an average error of 0.1% and an absolute average error of 4.1% for aluminum, and an average error of 5.1% and an absolute average error of 15.3% for stainless steel.

## 4. Conclusions

This paper presented a novel on-line estimation method for the depths of laser-drilled holes. Based on the laser-induced plasma region, transformed into a pixel value, we could obtain a correlation between the cumulative pixels and hole depth. Because the system could acquire and analyze images during laser machining in real time, the hole depth estimation was also in real time. The estimation mechanism based on a machine vision method could be combined with the depth control method for a conventional pulse laser to achieve more precise depth control for a laser-drilled hole.

## Figures and Tables

**Table 1.** Experiment parameters.

**Laser**				
**Laser Pump Energy**	**Radiation Energy**	**Pulse Duration**	**Pulse Repetition Rate**	**Wavelength**
18 J, 19 J, 20 J	145 mJ, 175 mJ, 200 mJ	6 ns	15 Hz	532 nm
**Workpiece Material**				
**Material**	**Focus Position**	**Thick**	**Ambient Temperature**	**Ambient Moisture**
Aluminum (Al, 5052)	Surface	0.6 mm	22 °C	60%
Stainless steel (SS, 420)		0.8 mm		
**Camera and Lens**				
**Camera Aperture**	**Camera Shutter Speed**	**Camera Object Distance**	**Camera Focus Length**	**Lens Focus Length**
16	117 μs	500 mm	75 mm	120 mm
